# Use of the ‘Accountability for Reasonableness’ Approach to Improve Fairness in Accessing Dialysis in a Middle-Income Country

**DOI:** 10.1371/journal.pone.0164201

**Published:** 2016-10-04

**Authors:** Mohammed Rafique Moosa, Jonathan David Maree, Maxwell T. Chirehwa, Solomon R. Benatar

**Affiliations:** 1 Division of Nephrology, Department of Medicine, Faculty of Medicine and Health Sciences, Stellenbosch University, Cape Town, South Africa; 2 Renal Unit, Tygerberg Academic Hospital, Cape Town, South Africa; 3 Biostatistics Unit, Centre for Evidence-based Health Care, Department of Interdisciplinary Health Sciences, Faculty of Medicine and Health Sciences, Stellenbosch University, Cape Town, South Africa; 4 Bioethics Centre, University of Cape Town, Cape Town, South Africa; University Medical Center Utrecht, NETHERLANDS

## Abstract

Universal access to renal replacement therapy is beyond the economic capability of most low and middle-income countries due to large patient numbers and the high recurrent cost of treating end stage kidney disease. In countries where limited access is available, no systems exist that allow for optimal use of the scarce dialysis facilities. We previously reported that using national guidelines to select patients for renal replacement therapy resulted in biased allocation. We reengineered selection guidelines using the ‘Accountability for Reasonableness’ (procedural fairness) framework in collaboration with relevant stakeholders, applying these in a novel way to categorize and prioritize patients in a unique hierarchical fashion. The guidelines were primarily premised on patients being transplantable. We examined whether the revised guidelines enhanced fairness of dialysis resource allocation. This is a descriptive study of 1101 end stage kidney failure patients presenting to a tertiary renal unit in a middle-income country, evaluated for dialysis treatment over a seven-year period. The Assessment Committee used the accountability for reasonableness-based guidelines to allocate patients to one of three assessment groups. Category 1 patients were guaranteed renal replacement therapy, Category 3 patients were palliated, and Category 2 were offered treatment if resources allowed. Only 25.2% of all end stage kidney disease patients assessed were accepted for renal replacement treatment. The majority of patients (48%) were allocated to Category 2. Of 134 Category 1 patients, 98% were accepted for treatment while 438 (99.5%) Category 3 patients were excluded. Compared with those palliated, patients accepted for dialysis treatment were almost 10 years younger, employed, married with children and not diabetic. Compared with our previous selection process our current method of priority setting based on procedural fairness arguably resulted in more equitable allocation of treatment but, more importantly, it is a model that is morally, legally and ethically more defensible.

## Introduction

End stage kidney disease is a major public health problem globally and its management is associated with an economic burden that is particularly onerous on low- and middle-income countries [1]. Worldwide, the incidence of end stage kidney disease is increasing at approximately 8% annually; global expenditure on maintenance dialysis in the decade spanning the late 1990s and early 2000s was an estimated US$ 1 trillion [2]. In 2013 the USA alone spent $30.9 billion treating patients with end stage kidney disease, accounting for 7.1% of the Medicare budget [3]. This expenditure by the USA solely on the treatment of end stage kidney disease patients exceeds the total health budget of all but the 29 richest countries in the world and closely matches the entire South African health budget of US$ 31.8 billion for 2013 [4].

Of the over 2 million people on renal replacement treatment worldwide, 90% are resident in high-income countries [5]. The correlation between a country’s level of prosperity and the prevalent dialysis population is now well established [6,7]. In low income countries—home to over 80% of the world’s population—less than 10% of patients with irreversible kidney failure have ready access to dialysis [8]. Where patients do have limited access the quality of dialysis is suboptimal [9]. The situation is gravest in sub-Saharan Africa, the poorest of all the world regions, where fewer than 1% of end stage kidney disease patients receive renal replacement treatment [10].

South Africa, a middle-income country, introduced renal replacement therapy in the 1960s, albeit on a limited basis. Faced with competing health care priorities, access to renal replacement treatment in South Africa is increasingly constrained and the country is grappling with ways to address the deteriorating situation [11]. In line with practice prevalent in wealthy countries before universal access to dialysis, an Assessment Committee at our institution formally assessed all patients with end stage kidney failure for enrollment to our renal replacement program. Until 1997 selection of patients was based on informal criteria taking into account social as well as medical factors that would optimize kidney allograft survival. Since 1997, following a national consensus meeting, the South African National Department of Health consolidated the criteria in use to develop a formal national policy on which we subsequently based our own criteria. We have reported that, on the basis of such decisions, only 47% of all patients with end stage kidney disease assessed by our committee over a 15-year period were accepted for renal replacement treatment. The likelihood of acceptance into the program was significantly biased in favor of patients who were employed, married, white and younger [12]. While we had shown that younger patients had better outcomes, [13], the racial disparity was a source of great consternation.

Faced with the moral dilemma of improving fairness in access to dialysis, and the need for an ethical basis for making decisions, our renal team used the accountability for reasonableness (A4R) approach to develop a hierarchical set of priorities in 2008. In the absence of a definitive basis in substantive justice for priority making decisions this ethical framework was one that has been designed to ensure procedurally fair decision-making [14,15].

## Material and Methods

The guidelines developed for prioritizing access to our renal replacement therapy program were based on the best available clinical evidence for good outcomes and the A4R principles of including relevant stakeholders, transparency, accountability through public access to the methods used, the availability of an appeals process for review of individual cases and dispute resolution, and openness to iteratively improve decision-making as new evidence became available. The team’s experience of local conditions and systems were also considered. The main premise of the guidelines for acceptance for dialysis remained the ultimate suitability of patients for, and optimum outcomes following kidney transplantation, as our resources do not permit the option of prolonged maintenance dialysis. The relevant stakeholders participating in drafting the guidelines included non-renal physicians, non-medical members of the renal team, laypersons, ethicists, lawyers, renal patients, hospital managers and social workers. The process was an iterative one and culminated in the Department of Health of the Western Cape Government formally adopting the policy and implementing it regionally [16].

The guidelines developed included a novel three-tiered hierarchy of priorities, based on the likelihood of best outcomes. Patients evaluated by the Committee were allocated to one of three categories, weighted by medical and social factors. Briefly, Category 1 patients were aged 50 years or less, had no significant comorbid disease and were eminently transplantable. Category 3 patients were aged over 60 years, or had severe comorbid disease that either precluded kidney transplantation or was associated with limited life expectancy. Category 2 patients comprised those with clinical and sociological characteristics between these two limits [16]. Importantly, the renal team concurrently negotiated an agreement with hospital management that all Category 1 patients would be dialyzed, even if the cap of 100 on the total number of patients on the dialysis program that serves as entry to the transplant program was exceeded. We also agreed that Category 2 patients would be dialyzed if resources were available at the time they required treatment. All Category 3 patients (and Category 2 patients who we could not accommodate because the program was saturated) would be cared for conservatively with compassion.

Our teaching hospital based Renal Unit is one of two tertiary referral centers serving adults with kidney disease in Western Cape Province; we are responsible for the care of approximately one-half of the Western Cape population estimated at 6.1 million (Statistics South Africa, 2014, Pretoria, Statistical Release P0302). The Assessment Committee evaluated a total of 1101 patients with stage 5 chronic kidney disease in the seven year period for enrollment into our renal replacement treatment program. The mean age of the patients assessed was 41.9 years (standard deviation ±12.6), and 585 (53.1%) were males. The racial profile generally reflected that of the population of the Western Cape: mixed-race 702 (63.8%), black 309 (28.1%), white 75 (6.8%), and foreign nationals 15 (1.4%). The Assessment Committee met once weekly and comprised the attending physicians, nephrologists, social worker, hospital manager and dialysis staff. Attempts at involving representatives of the community were unsuccessful, as they felt daunted by the whole process and reluctant to accept the responsibility for making these life or death decisions. For each patient evaluated medical and social workers’ reports were presented. The Assessment Committee deliberated the merits of each case based on the guidelines that had been developed and allocated each patient to one of three categories described. The Category 3 and those Category 2 patients not accepted and/or their families were informed of the committee’s decision soonest possible, with reasons provided for the decision. Patients not placed were counseled and provided with all necessary medical care to ensure maximum comfort and minimize any suffering; where necessary patients were seen by the social worker and financial support provided; hospice care was arranged if required. Alternative options were discussed with patients and their families, including the possibility of review and of treatment in the private sector. All decisions were recorded, with reasons.

We reviewed data of all patients evaluated by the Assessment Committee. The data were collected over the seven-year period included demographic information, social habits, cause of chronic kidney failure, comorbid diseases, and social information including employment, marital status and number of dependent children, domicile, the decision of the Assessment Committee, and documented reasons why patients were refused treatment. The study reviewed data collected from May 1, 2008 to April 30, 2015. Data is presented in one-year intervals from the commencement date.

Stata version 13.1 (StataCorp, 2013) statistical software was used to compute summary statistics and associations between acceptance and other patient covariates. Summary statistics are reported as means with standard deviation for continuous data, and frequencies with percentages for categorical data. Continuous data were analyzed using Student’s t-test and categorical data using the chi-square test. Logistic regression was applied to obtain odds ratios for acceptance comparing different categories of categorical variables such as employment and unit change in continuous variables such as age. The confounding effect of patient characteristics was adjusted through multivariate logistic regression. For each covariate included in multivariate logistic regression, the odds ratio and associated 95% confidence intervals are reported. Likelihood ratio test was employed to identify the best parsimonious subset of characteristics with high predictive power. The association between patient characteristics and acceptance was considered statistically significant if the p-value was less than 5%. To evaluate transplant outcomes, acturial one-year patient and graft survival rates and their associated 95% confidence intervals were computed. Graft survival was censored for patient mortality.

Approval to conduct the study was granted by the Human Research Ethics Committee of Stellenbosch University (N14/04/028). Waiver of consent was approved by the Committee because of the challenge of obtaining consent retrospectively. Data were anonymized and de-identified before analysis and was reported in aggregate.

## Results

**Fig 1:**
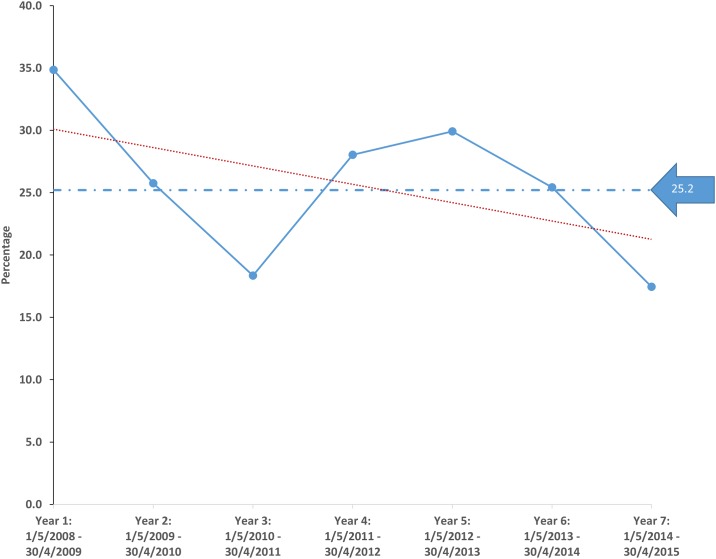
The total number of patients assessed over the period of the study within each assessment category. The majority of patients were assessed as Category 2, which meant that they could be offered treatment only if facilities were available at the time they required dialysis.

**Fig 2:**
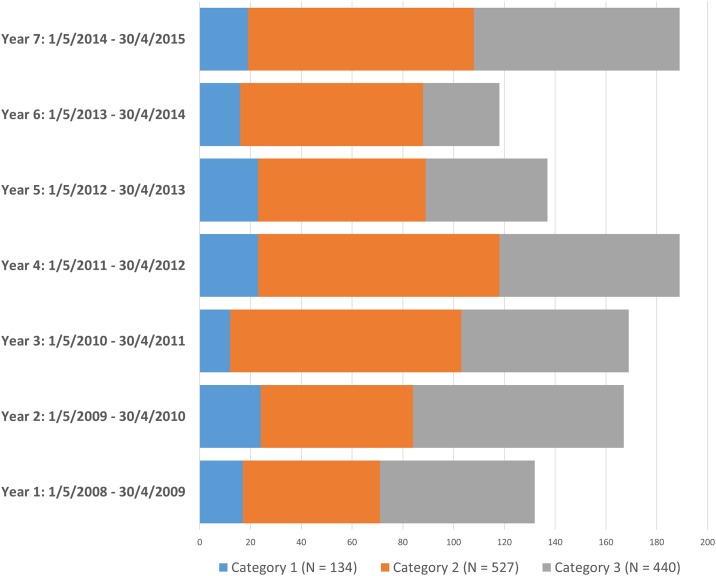
Patient treatment acceptance rates over the seven year time period. The overall acceptance rate was 25.2% (stippled line) with the trend being downward (dotted line). Acceptance almost halved in the seventh year compared to the first, from 34.8% to 17.5%. The fluctuations in numbers with increases in Years 4 and 5 were related to slight expansions in the renal replacement program—the capped number of patients we were allowed to treat was increased from 100 to 120.

Over the seven-year period of this study the number of end stage kidney failure patients assessed by the Assessment Committee fluctuated but with a trend to increase: the total number of patients ranged from 118 in Year 6 to 189 in Year 7 averaging 157.3 patients annually (Fig 1). Of the 1101 patients assessed in this period only 277 (25.2%) were accepted for renal replacement treatment; the acceptance rates fluctuated over the time period with a downward trend (Fig 2). Of all the patients assessed 527 (47.9%) were placed in Category 2 followed by 440 (40.0%) in Category 3 and 134 (12.2%) in Category 1. The relative proportion of patients in the three assessment categories did not vary significantly over the seven-year period (Fig 1). Comparative details of patients in the three categories as well as those accepted and treated conservatively are shown in Table 1. Of the Category 1 patients 132 (98.5%) were accepted for treatment and 438 (99.5%) Category 3 patients were denied treatment. Of those classified as Category 2, only 142 (26.9%) could be accommodated on the treatment program. There was a slight male preponderance (53.0%) of the overall number of patients assessed but significantly more women were accepted for treatment (149, 28.8%) compared to men (127, 21.7%). Two patients in Category 1 who were eligible for dialysis treatment elected to receive their treatment in the private sector, whereas two patients in Category 3 who were refused treatment, requested that dialysis be initiated after giving guarantees that they would continue treatment in the private sector within an agreed period. Diabetes mellitus was significantly more common as a cause of end stage kidney failure in the patients refused treatment and less than 4% of patients with human immunodeficiency virus (HIV) infection were accepted. Of the hepatitis B virus infected patients assessed, only 1 (4%) was accepted for treatment. The distance that a patient resided from a dialysis unit or whether the patient resided in an urban or rural area, did not influence the decision to accept a patient.

**Table 1:** Details of the 1101 patients in the three assessment categories compared with each other and the details of patients accepted for renal replacement treatment compared to those treated conservatively.

	Category 1	Category 2	Category 3	*P-value*	Patients for dialysis	Patients for palliation	OR	*P-Value*
	(N = 134)	(N = 527)	(N = 440)		(N = 276)	(N = 825)		
Mean age, years (±SD)	33.3±10.7	40.7±11.1	46.0±13.2	<0.001	35.8 (±10.9)	44.0 (±12.4)	0.9	<0.001
Male, n (%)	71 (53.0)	267 (50.7)	247 (56.1)	0.236	127 (46.0)	458 (56.0)	0.7	0.006
HIV infected, n (%)	0 (0)	43 (8.2)	60 (13.6)	<0.001	4 (1.4)	99 (12.0)	0.1	<0.001
HBV infected, n (%)	0 (0)	9 (1.7)	16 (3.0)	0.023	1 (0.4)	24 (2.9)	0.1	0.039
Smoker n, (%)	24 (17.9)	141 (27.0)	104 (23.6)	0.006	49 (17.8)	220 (26.7)	0.6	0.008
**Race, n (%)**				**0.004**				
African	25 (18.7)	165 (31.3)	119 (27.1)		64 (23.2)	245 (29.7)	REF	
Mixed race	98 (73.1)	324 (61.5)	280 (63.6)		189 (68.5)	513 (62.2)	1.4	0.036
White	11 (8.2)	35 (6.6)	29 (6.6)		23 (8.3)	52 (6.3)	1.7	0.067
Foreign nationals	0 (0.0)	3 (0.6)	12 (2.7)		0 (0.0)	15 (1.8)	-	-
**Marital status, n (%)**				**<0.001**				
Ever married	64 (47.8)	277 (52.6)	198 (45.0)		152 (55.0)	387 (47.0)	1.1	0.445
Never married	67 (50.0)	216 (41.0)	159 (36.1)		115 (42.0)	327 (40.0)	REF	
Not documented	3 (2.2)	34 (6.5)	83 (18.9)		9 (3.3)	111 (13.5)	0.2	<0.001
**Dependents** (median (range))	1 (0–6)	1 (0–8)	0 (0–9)	**<0.001**^a^	1 (0–7)	0 (0–9)	1.3	<0.001
Not documented	4	65	136		14	191		
**Domicile, n (%)**				**0.338**				
Metropole	114 (85.0)	463 (87.9)	373 (84.7)		243 (88.0)	707 (85.7)	REF	
Rural	20 (14.9)	64 (12.1)	65 (14.8)		33 (12.0)	116 (14.1)	0.8	0.37
Not documented	0 (0)	0 (0)	2 (0.5)		0 (0)	2 (0.2)	-	
**Education, n (%)**				**<0.001**				
Illiterate /Primary	16 (11.9)	114 (21.6)	82 (18.6)		31 (11.2)	181 (22.0)	REF	
Secondary /Tertiary	103 (76.9)	298 (56.5)	175 (39.8)		204 (74.0)	372 (45.1)	3.2	<0.001
Not documented	15 (11.2)	115 (22.0)	183 (41.6)		41 (14.9)	272 (33.0)	0.9	0.619
**Employment status**				**<0.001**				
Employed	119 (88.8)	240 (45.5)	150 (34.1)		214 (78.0.)	298 (36.1)	4.2	<0.001
Pension	0 (0.0)	26 (5.0)	51 (11.6)		1 (0.4)	76 (9.2)	0.1	<0.012
Unemployed	12 (9.0)	223 (42.3)	149 (33.9)		56 (20.3)	328 (40.0)	REF	
Not documented	3 (2.2)	38 (7.2)	90 (20.4)		6 (2.2)	125 (15.2)	0.3	0.004
**Distance n (%)**				**0.784**				
Less than 100 km	114 (85.1)	463 (87.9)	373 (84.8)		243 (88.0)	707 (85.7)	REF	
100–150 km	10 (7.5)	35 (6.6)	37 (8.4)		19 (6.89)	63 (7.6)	0.9	0.631
150–200 km	5 (3.7)	15 (2.8)	14 (3.2)		6 (2.2)	28 (3.4)	0.6	0.300
More than 200 km	5 (3.7)	14 (2.7)	14 (3.2)		8 (2.9)	25 (3.0)	0.9	0.863
Not documented	0	0	2 (0.5)		0 (0.0)	2 (0.2)	-	-
**Primary renal disease, n (%)**				**<0.001**				
Diabetes mellitus	3 (2.2)	67 (12.7)	90 (20.5)		11 (4.0)	149 (18.1)	0.2	<0.001
Others	131 (97.8)	460 (87.2)	350 (79.6)		266 (96.4)	678 (82.2)	REF	

^a^ Post hoc analysis: Patients in Category 3 has higher number of dependents compared to 1 and 2; OR, odds ratio; SD, standard deviation; REF, reference set; HIV, human immunodeficiency virus.; HBV, hepatitis B virus.

By the end of April 2016, marking one year after the completion of patient entry into the study, 91 (33.0%) of the patients accepted for treatment had received a primary kidney transplant; of these 48 (17.4%) received their new kidneys within one year. Over the same time period, 4 (1.4%) received a second transplant and 49 (17.8%) died before they could be transplanted. Living donors were the source of 63 (69.2%) of the kidneys transplanted and deceased donors for 28. In terms of transplant outcomes, the actuarial one-year patient and graft survival rates of 92% (95% CI: 84%–96%) and 80% (95% CI: 70%–87%) respectively, with the latter censored for death.

On multivariate analysis, the odds of receiving treatment was greater for those who were younger, married, female, had more dependent children, had higher levels of education and were employed (Table 2). Diabetes reduced the odds of patients being offered treatment by 88%. Only 6.9% of all diabetics assessed were accepted for dialysis. Patients with HIV infection or hepatitis B infection had 95% and 90% lower odds, respectively, of being treated compared to those uninfected. Race did not impact on the decision to accept patients for treatment.

**Table 2:** Multivariate logistic regression analysis of factors impacting on acceptance for renal replacement treatment.

Variable (N = 892) ^a^	Odds Ratio (95% CI)	P-value
Age	0.92 (0.90–0.94)	<0.001
Gender: male	0.61 (0.43–0.88)	0.007
HIV infected	0.05 (0.02–0.14)	<0.001
Diabetic	0.22 (0.10–0.46)	<0.001
HBV infected	0.10 (0.01–0.94)	0.044
Marital status		
Never married	REF	
Ever married	3.34 (2.06–5.40)	<0.001
Dependents	1.35 (1.16–1.57)	<0.001
Educational level		
Illiterate/Primary	REF	
Primary/ Tertiary	3.32 (2.01–5.50)	<0.001
Not documented	3.19 (1.63–6.23)	0.001
Employment		
Unemployed	REF	
Employment	4.92 (3.25–7.43)	<0.001
Pension	0.18 (0.02–1.41)	0.103
Not documented	1.62 (0.45–5.87)	0.465
Smoking		
No	REF	
Yes	0.55 (0.36–0.86)	0.009
Not documented	2.01 (1.26–3.20)	0.003

^a^ Patients with non-missing data on all the variables included in multivariate analysis; CI, 95% confidence interval; REF, reference set. HIV, human immunodeficiency virus; HBV, hepatitis B virus.

The reasons for refusing patients were broadly divided into medical and socioeconomic (Table 3). The primary reason that a patient was excluded by the committee was documented; under primary reasons, medical issues were more common than socioeconomic ones. Additional factors contributed in most cases and when the reasons were aggregated socioeconomic reasons were more common than medical ones. The fact that a patient was economically dependent was the most common socioeconomic factor in the primary as well the aggregated reasons patients were turned down. Under the medical conditions, diabetes mellitus was the most common reason for refusal and accounted for almost one-quarter of all primary medical exclusions, followed by age over 60 years and HIV infection. The remaining listed medical exclusions accounted for less than 10% each. Under socioeconomic factors, poor adherence to treatment and drug/alcohol addiction were the most common psychosocial primary reasons for exclusion. Socioeconomic factors generally associated with reduced ability to comply or ability to benefit optimally, included extreme poverty and lack of gainful employment necessitating basic subsistence social grants provided by the state (that allow living at US$2 per day). Severe obesity (body mass index greater than 35 kg/m^2^), cardiovascular disease and hepatitis B virus infections were uncommon reasons for exclusions, each accounting for fewer than 5% of patients refused treatment.

**Table 3:** Reasons for patients being refused treatment listed with an aggregate of the individual items.

	Primary	Secondary	Tertiary Plus	Aggregate^a^
	(N = 825)	(N = 631)	(N = 236)	(N = 1694) (%)
**Medical**	**n = 447 (%)**	**n = 249 (%)**	**n = 73 (%)**	**n = 769**
Malignancy	7 (1.6)	8 (3.2)	0 (0.0)	15 (0.9)
Cardiovascular disease	36 (8.1)	21 (8.4)	6 (8.2)	63 (3.7)
Diabetes	110 (24.6)	48 (19.3)	6 (8.2)	164 (9.7)
Age > 60 years	86 (19.2)	57 (22.9)	14 (19.2)	157 (9.3)
HIV infection	74 (16.6)	18 (7.2)	6 (8.2)	98 (5.8)
Other	64 (14.3)	61 (24.5)	31 (42.5)	156 (9.2)
Obese	37 (8.3)	22 (8.8)	3 (4.1)	62 (3.7)
Hepatitis B virus infection	16 (3.6)	5 (2.0)	3 (4.1)	24 (1.4)
Late presentation	17 (3.8)	9 (3.6)	4 (5.5)	30 (1.8)
**Sociological**	**n = 378 (%)**	**n = 382 (%)**	**n = 165 (%)**	**n = 925**
Unemployed	56 (14.8)	36 (9.4)	22 (13.3)	114 (6.6)
Home circumstances/Distance	8 (2.1)	17 (4.5)	14 (8.5)	39 (2.3)
Poor adherence	60 (15.9)	34 (8.9)	17 (10.3)	111 (6.6)
Lack of dependent children	34 (9.0)	119 (31.2)	59 (35.8)	212 (12.5)
Not breadwinner	115 (30.4)	107 (28.0)	25 (15.2)	247 (14.6)
Drug dependence	58 (15.3)	48 (12.6)	22 (13.3)	128 (7.6)
Foreign nationals	12 (3.2)	2 (0.5)	0 (0.0)	14 (0.8)
Other	35 (9.3)	19 (5.0)	6 (3.6)	60 (3.5)

^a^ In this column the percentages are given for the aggregate

Of the 825 patients selected for conservative treatment, 58 (7%) launched appeals against the decision. On review the initial decision was upheld in all but three patients (94.5%).

## Discussion

Physicians practicing nephrology in low- and middle-income countries face challenges that are significantly different from those experienced by their counterparts in well-resourced countries [1]. The former are faced with a burgeoning population of patients with end stage kidney disease as a result of aging and the diabetes mellitus epidemic, beside those chronic kidney diseases caused by infectious diseases still prevalent here [17,18]; in high income regions, on the other hand, the rising incidence of chronic kidney diseases seen in the last four decades of the last century has stabilized and the last decade has seen a decline in the incidence of the disease in all age groups [19]. This places undue hardships on economies already struggling under the yoke of the global economic recession further widening heath disparities [20]. Few low- and middle-income countries have universal access to renal replacement therapy and patients often have to make out-of-pocket payments for treatment, often leaving families destitute [21]. Where state funding for uninsured patients is restricted it is unclear how patients are selected for treatment [22,23]. In South Africa, the state supports a limited number of patients on renal replacement treatment but in the face of other health priorities, the situation for patients with end stage kidney diseases is deteriorating. In our unit the number of patients with irreversible kidney disease to whom we were unable to offer treatment increased from 52.7% in 2006 [12] to almost 75% as reported here. The rising rejection rate is a function of several factors. There are an increasing number of end stage kidney disease patients requiring renal replacement treatment. Using available information on the Western Cape Province’s population of 6.1 million (Statistics South Africa, 2014, Pretoria, Statistical release 1-8-2014), the estimated incidence of end stage kidney disease in South Africa of 250 per million population [24] and our average transplant and dialysis attrition rates, we have estimated that we currently meet a mere 7.8% of the Western Cape Province’s dialysis needs, substantially down from the 12.5% at the start of this project in 2008. The situation is aggravated by a steady decline in the number of kidney transplants performed in our unit. Reasons for the falling rate of kidney transplants are likely multifactorial, including changing public awareness and attitudes because of being poorly informed [25]; a change in the provincial government head injury policy which required head injury patients with very poor prognosis be managed at the peripheral hospital and not to be referred to our tertiary institution; the immense burden of trauma means that emergency unit staff prioritize saving those who are salvageable and passing over opportunities to refer potential donors because staff and resource constraints [11]; competition for organs with the private sector with whom we now share; and bad publicity arising from the sale of organs scandal [26,27].

The still unraveling global economic crisis has impacted particularly severely on developing countries like South Africa. These economic realities translate into limited resources being available for health care, especially relatively costly treatments such as dialysis. South Africa has a two-tiered health system with 16% of the population insured and guaranteed dialysis while the remaining 84% relies almost solely on the state for the provision of health services and hence for access to dialysis and transplantation [28,29]. Our Renal Unit almost exclusively services the latter, largely indigent population. In terms of resources the total number of patients we are permitted to treat is capped by the management of the hospital, with the capped number increasing from 100 to 120 patients over the period of this study. This places an enormous moral and ethical burden on the shoulders of the clinical staff responsible for managing these patients. As nephrologists caring for these patients, we have to decide how best to use ever-dwindling health care resources and severely stretched tertiary healthcare services, with consideration of both common-good outcomes and individual rights. This balance is arguably appropriate when spending from a public purse and in the context of limited resources. As previously argued “…the challenge is to create harmony between the rights of individuals and responsibility to society in ways that promote both individuality and solidarity and that also sustain the moral integrity of caring health professionals” [30]. There are no simple solutions to such complex challenges. A test case regarding the ‘right to life’ from renal support therapy taken to the South African Constitutional Court led to a decision that supported the principles embraced by the A4R process, thus adding indirect support for this approach [31].

Against this background a priority-setting process has become critically important with the challenge being to ensure the most fair, equitable and transparent selection of patients for access to an increasingly scarce resource [32]. In contrast to other allocative processes that rely more predominantly on an economic approach, the A4R framework employs an ethical approach that emphasizes the procedural fairness of rationing decisions. Since it is often difficult to agree on *what* decisions are made in the absence of any agreed upon substantive base for distributive justice, it is more likely consensus can be achieved among relevant stakeholders on *how* decisions are made. The A4R framework requires the fulfillment of four procedural conditions: relevance to the context in which they are made; transparency relating to the reasons for the decisions, accountability through accessibility for public scrutiny, and openness to challenge or review and leadership/regulatory mechanisms to ensure that these conditions are met [33,34]. Gibson and her colleagues have argued for a fifth condition, ‘empowerment’, to allow for more effective participation of various stakeholders [34]. To eliminate all bias Marckmann and his colleagues [35], have added consistency, participation and managing conflict of interests to the list of conditions. There is ongoing refinement of the concept of A4R process and further research will no doubt be required to test its value and robustness under various conditions [36].

Our revised guidelines reversed the white racial predominance of patients selected we had previously reported [13], but the predominance of women under the current guidelines is an unforeseen and unintended outcome. This may be related to mothers playing the main child-rearing role in single parent households, which are common in the impoverished communities we serve. Other biases continue to exist: the preponderance of patients reported here who are married, have children, more highly educated and are employed, and may reflect the persistence of pragmatic, utilitarian considerations among members of the Assessment Committee. Two main groups of factors militated against patients being enrolled for treatment: associated medical conditions least favorable to maximizing kidney transplant survival and poor socioeconomic conditions that reduce the ability to comply and the potential for good outcomes. Compared to our previous report there has been a salutary decline in rejection of patients primarily on socioeconomic criteria but these continued to contribute as factors considered in decision-making.

In redeveloping the guidelines [16], maintaining the age threshold of 60 years attracted some controversy. Our patients are considerably younger than patients commenced on dialysis in high income countries; this relates to the main etiologies of chronic kidney disease being mainly infective in our population compared to diabetes mellitus in wealthier nations [3,11]. While there is conflicting evidence in the literature of the impact of age on graft and patient survival [37,38], our decision was supported by data including our own, that increasing age was associated with poorer graft survival and higher patient mortality [39]. The preference for younger patients for dialysis and transplantation has always been a sad reality ever since dialysis became available [40]. Rationing on the basis of age recognizes that the youngest have the most to lose in terms of life-years and allocation of resources in a ‘youngest first’ approach is in line that with favoring the worst off, one of eight important principles in allocating scarce resources [41]. Support for using age as a criterion limiting scarce treatment has been sanctioned by prominent bioethicists including Daniels and Callahan [42,43], with others having opposing views [44,45]. Obesity, on the increase globally, affects 13.5% of South African men and 42% of adult women across all ethnic groups [46]. Obesity excluded less than 10% of our assessed patients from treatment; it is associated with higher acute graft rejection rates and poorer graft survival although, paradoxically, obese patients survive better on dialysis [47–49]. Being diabetic significantly reduced the odds of acceptance for treatment; in our experience—shared by others—diabetics fare poorly following kidney transplantation. Compared to non-diabetics, diabetics are older, fatter, have more underlying cardiovascular disease and after transplantation more often succumb to cardiovascular disease and infections [50]. Others, though, have reported more favorable experiences [51,52].

HIV infection reduced the odds of acceptance by 95% with only four patients entering our program. Our reservation in accepting HIV-positive patients relates to limited experience with kidney transplantation in these patients, although promising results have recently been reported [53,54]. However, serious challenges remain in managing these patients including the high risk of acute rejection [55]. Hepatitis B viral infections are associated with adverse renal transplant outcomes [56,57] and we are unable to afford the nucleoside/nucleotide analogues that have dramatically improved the outcomes of renal transplantation in infected patients [58,59]. Active alcohol and recreational drug dependency excluded more than 15% of patients; alcohol use is an important cause of non-adherence with immunosuppressive regimen [60]. Smoking has been well documented to have a detrimental influence on patient and graft survival [61–63]. The Western Cape population is largely rural and consequently late presentation and poor adherence are frequent challenges [64]. Studies confirm that the need for urgent dialysis is associated with higher morbidity and mortality and the prolongation of hospital stay adds to the costs of treating patients presenting late for treatment [65].

A strategy to select patients, such as the one we are proposing, has the potential to maximize use of scarce resources and minimize the risk of further impoverishing desperate families. At the same time our approach allows patients who are accepted for treatment to enjoy high quality medical care. The A4R process we employed was a participative one that led to the development of procedurally fair guidelines and a novel approach to categorizing patients. Prior to the new guidelines, clinicians carried full responsibility for all decisions, even though resource limitation—an economic consideration—was the major factor limiting access to treatment. With the new approach, clinical managers responsible for the hospital budget participate in the priority setting process, thus sharing the moral burden of these difficult decisions that are made within the framework of an institutionally sanctioned priority setting process. To our knowledge this is the first report of the application of the A4R framework to assist in prioritizing access to dialysis. There are reports of the use of the A4R process in other situations in low-income countries, the most notable being the Response to Accountable Priority Setting for Trust in Health Systems (REACT) study launched in 2006 in three central African countries to improve health outcomes at district level [66,67]. Models of priority setting that rely on technical approaches such as cost-effectiveness and capacity considerations have often proven unsatisfactory and have resulted in a conflict in values such as between equity and efficiency [67]. It is reassuring to know that the broad approach we have adopted aligns with recent priority settings recommendations made for application at the micro-, meso- and macrolevels of health care delivery [68,69]. It is also reassuring that the early outcomes delivered in terms of patient and graft survival following transplantation are comparable to the outcomes in other low- and middle income countries [70], justifying our procedural approach to the use of the scarce resources to optimumbenefit.

As chronic kidney disease becomes an increasingly greater public health challenge, the need for priority setting seems unavoidable, even among the richest of nations [71,72]. Developing a clear policy through an evidence based, accountable, transparent and contestable process in which all stakeholders participate and that takes into consideration relevant values, including legitimate competing ones, will make it easier to implement and defend morally, ethically and legally [14,73].

## Conclusion

We have presented a new model of hierarchical priority setting, developed using the A4R framework. The innovation of weighting components in the guidelines has enhanced our decision-making process. Our model’s greatest success was in ensuring that all patients who were “ideal” candidates would receive treatment. However, it failed to completely eliminate inequity related to gender and social circumstances and we will therefore have to continue to review our guidelines and seek ways to address these challenges. We hope that our experience will serve as an incentive for others, especially those in resource-constrained environments to review their priority setting practices and take up the challenge of developing their own guidelines using this values-based approach.

## References

[1] White SL, Chadban SJ, Jan S, Chapman JR, Cass A. How can we achieve global equity in provision of renal replacement therapy? Bull World Health Organ. 2008; 86: 229–237. S0042-96862008000300017 [pii]. 10.2471/BLT.07.041715 18368211 PMC2647401

[2] Lysaght MJ. Maintenance dialysis population dynamics: current trends and long-term implications. J Am Soc Nephrol. 2002; 13 Suppl 1: S37–S40. 11792760

[3] United States Renal Data System 2015 USRDS annual data report: Epidemiology of kidney disease in the United Sates. 2015 National Institutes of Health, National Institute of Diabetes and Digestive and Kidney Diseases, Bethesda, MD, 2015.

[4] The World Bank Total Health Expenditure. 2015. Available: (http://data.worldbank.org/indicator/SH.XPD.TOTL.ZS). Accessed 16 June 2016.

[5] Nugent RA, Fathima SF, Feigl AB, Chyung D. The burden of chronic kidney disease on developing nations: a 21st century challenge in global health. Nephron Clin Pract. 2011; 118: c269–c277. 000321382 [pii]; 10.1159/000321382 21212690

[6] Jha V, Garcia-Garcia G, Iseki K, Li Z, Naicker S, Plattner B, et al Chronic kidney disease: global dimension and perspectives. Lancet 2013; 382: 260–272. S0140-6736(13)60687-X [pii]; 10.1016/S0140-6736(13)60687-X 23727169

[7] Grassmann A, Gioberge S, Moeller S, Brown G. ESRD patients in 2004: global overview of patient numbers, treatment modalities and associated trends. Nephrol Dial Transplant. 2005; 20: 2587–2593. 10.1093/ndt/gfi159 16204281

[8] Eggers PW. Has the incidence of end-stage renal disease in the USA and other countries stabilized? Curr Opin Nephrol Hypertens. 2011; 20: 241–245. 10.1097/MNH.0b013e3283454319 21422925

[9] Kher V. End-stage renal disease in developing countries. Kidney Int. 2002; 62: 350–362. kid426 [pii]; 10.1046/j.1523-1755.2002.00426.x 12081600

[10] Bamgboye EL. Barriers to a functional renal transplant program in developing countries. Ethn Dis 2009; 19: S1–S9. 19484877

[11] Moosa MR, Meyers AM, Gottlich E, Naicker S. An effective approach to chronic kidney disease in South Africa. S Afr Med J. 2016; 106: 156–159. 10.7196/SAMJ.2016.v106i2.9928 26821893

[12] Moosa MR, Kidd M. The dangers of rationing dialysis treatment: the dilemma facing a developing country. Kidney Int. 2006; 70: 1107–1114. 5001750 [pii]; 10.1038/sj.ki.5001750 16883316

[13] Moosa MR. Impact of age, gender and race on patient and graft survival following renal transplantation—developing country experience. S Afr Med J. 2003; 93: 689–695. 14635558

[14] Martin D, Singer P. A strategy to improve priority setting in health care institutions. Health Care Anal. 2003; 11: 59–68. 10.1023/A:1025338013629 14510309

[15] Daniels N, Sabin J. The ethics of accountability in managed care reform. Health Aff. (Millwood) 1998; 17: 50–64. 10.1377/hlthaff.17.5.50 9769571

[16] Moosa MR. Guideline: Priority setting approach in the selection of patients in the public sector with end-stage kidney failure for renal replacement treatment in the Western Cape Province; 2013. Available: http://s3.documentcloud.org/documents/19489/moosa-priority-setting-policy-final-feb-24-2010-final.pdf. Accessed 10 June 2016

[17] Barsoum RS. End-stage renal disease in North Africa. Kidney Int Suppl. 2003; S111–S114. kid8322 [pii]; 10.1046/j.1523-1755.63.s83.23.x 12864887

[18] Shaheen FA, Al-Khader AA. Preventive strategies of renal failure in the Arab world. Kidney Int Suppl. 2005; S37–S40. KID9807 [pii]; 10.1111/j.1523-1755.2005.09807.x 16108969

[19] Murphy D, McCulloch CE, Lin F, Banerjee T, Bragg-Gresham JL, Eberhardt MS, et al Trends in prevalence of chronic kidney disease in the United States. Trends in prevalence of chronic kidney disease in the United States. Ann Internl Med. 2016; 10.7326/M16-0273 27479614 PMC5552458

[20] Benatar SR, Gill S, Bakker I. Global health and the global economic crisis. Am J Public Health. 2011; 101: 646–653. AJPH.2009.188458 [pii]; 10.2105/AJPH.2009.188458 21330597 PMC3052329

[21] Sakhuja V, Sud K. End-stage renal disease in India and Pakistan: burden of disease and management issues. Kidney Int Suppl. 2003; S115–S118. kid8323 [pii]; 10.1046/j.1523-1755.63.s83.24.x 12864888

[22] Garcia-Garcia G, Monteon-Ramos JF, Garcia-Bejarano H, Gomez-Navarro B, Reyes IH, Lomeli AM, et al Renal replacement therapy among disadvantaged populations in Mexico: A report from the Jalisco Dialysis and Transplant Registry (REDTJAL). Kidney Int. 2005; 68: S58–S61. 10.1111/j.1523-1755.2005.09710.x 16014102

[23] Sakhuja V, Kohli HS. End-stage renal disease in India and Pakistan: incidence, causes, and management. Ethn Dis. 2006; 16: S2–S3. 16774005

[24] Naicker S, Bello AK, El Nahas M. Chronic kidney diseases: Focus on Africa In: El Nahas M, editor. Kidney diseases in the developing world and ethnic minorities. London: Taylor and Francis 2005; pp. 137–160. 10.1201/b14128-8

[25] Etheredge HR, Turner RE, Kahn D. Public attitudes to organ donation among a sample of urban-dwelling South African adults: a 2012 study. Clin Transplant. 2013; 27: 684–692. 10.1111/ctr.12200 23968357

[26] Bass D. Kidneys for cash and egg safaris—can we allow 'transplant tourism' to flourish in South Africa? S Afr Med J. 2005; 95: 42–44. 15762247

[27] Scheper-Hughes N. Keeping an eye on the global traffic in human organs. Lancet. 2003; 361: 1645–1648. 10.1016/S0140-6736(03)13305-3 12747896

[28] Kevany S, Benatar SR, Fleischer T. Improving resource allocation decisions for health and HIV programmes in South Africa: Bioethical, cost-effectiveness and health diplomacy considerations. Glob Public Health 2013; 8: 570–587. 10.1080/17441692.2013.790461 23651436

[29] Mayosi BM, Benatar SR. Health and health care in South Africa—20 years after Mandela. N Engl J Med. 2014; 371: 1344–1353. 10.1056/NEJMsr1405012 25265493

[30] Benatar S. Facing ethical challenges in rolling out antiretroviral treatment in resource-poor countries: comment on "They call it 'patient selection' in Khayelitsha. Camb Q Health Ethics 2006; 15: 322–330. 10.1017/S0963180106060427 16862936

[31] [Anonymous] (1998) Soobramoney v. Minister of Health, Kwa-Zulu Natal. (1998). (1) SA 765 (South African Constitutional Court).

[32] Benatar SR. Health Care Reform and the Crisis of HIV and AIDS in South Africa. N Engl J Med. 2004; 351: 81–92. 10.1056/NEJMhpr033471 15229313

[33] Daniels N. and Sabin J. Setting Limits Fairly: Can We Learn to Share Medical Resources? Oxford: 2002; Oxford University Press 10.1093/acprof:oso/9780195149364.001.0001

[34] Gibson J, Mitton C, Martin D, Donaldson C, Singer P. Ethics and economics: does programme budgeting and marginal analysis contribute to fair priority setting? J Health Serv Res Policy. 2006; 11: 32–37. 10.1258/135581906775094280 16378530

[35] Marckmann G, Schmidt H, Sofaer N, Strech D. Putting public health ethics into practice: A systematic framework. Frontiers in Public Health. 2015; 3:23, e1–e8. 10.3389/fpubh.2015.00023 25705615 PMC4319377

[36] Benatar SR, Ashcroft R. International perspectives on resource allocation Reference Module in Biomedical Sciences. Elsevier 2015; 10.1016/B978-0-12-801238-3.92830-7

[37] Faravardeh A, Eickhoff M, Jackson S, Spong R, Kukla A, Issa N, et al Predictors of graft failure and death in elderly kidney transplant recipients. Transplantation. 2013; 96: 1089–1096. 10.1097/TP.0b013e3182a688e5 24056622

[38] Fabrizii V, Winkelmayer WC, Klauser R, Kletzmayr J, Säemann MD, Steininger et al Patient and graft survival in older kidney transplant recipients: Does age matter? J Am Soc Nephrol. 2004; 15: 1052–1060. 10.1097/01.ASN.0000120370.35927.40 15034109

[39] Saudan P, Berney T, Leski M, Morel P, Bolle JF, Martin PY. Renal transplantation in the elderly: a long-term, single-centre experience. Nephrol Dial Transplant. 2001; 16: 824–828. 10.1093/ndt/16.4.824 11274281

[40] Alexander S. They Decide Who Lives, Who Dies: Medical miracle and a moral burden of a small committee. Life: 1962; 53 September 9: 102–125.

[41] Persad G, Wertheimer A, Emanuel EJ. Principles for allocation of scarce medical interventions. Lancet. 2009; 373: 423–431. 10.1016/S0140-6736(09)60137-9 19186274

[42] Callahan D. D. Setting limits: medical goals in an aging society. Washington, DC: Georgetown University Press; 1995.

[43] Daniels Norman. Am I my parents' keeper? An essay on justice between the young and the old. Oxford University Press; 1988.

[44] Williams A, Evans JG. The rationing debate: Rationing health care by age. BMJ. 1997; 314: 820 10.1136/bmj.314.7083.820 9081009 PMC2126190

[45] Rutecki GW. Would treatment allocation according to age-contingent depreciation be ethical? A dialysis and transplantation paradigm. Ethic Med. 2016; 27: 99–107.

[46] Ng M, Fleming T, Robinson M, Thomson B, Graetz N, Margono C, et al Global, regional, and national prevalence of overweight and obesity in children and adults during 1980–2013: a systematic analysis for the Global Burden of Disease Study 2013. Lancet. 2014; 384: 766–781. S0140-6736(14)60460-8 [pii]; 10.1016/S0140-6736(14)60460-8 24880830 PMC4624264

[47] Curran SP, Famure Y, Li Z, Kim SJ. Increased recipient body mass index is associated with acute rejection and other adverse outcomes after kidney transplantation. Transplantation 2014; 97: 64–70. 10.1097/TP.0b013e3182a688a4 24056619

[48] Aalten J, Christiaans MH, de Fijter H, Hene R, van der Heijde JH, Roodnat J, el at The influence of obesity on short- and long-term graft and patient survival after renal transplantation. Transpl Int. 2006; 19: 901–907. TRI367 [pii]; 10.1111/j.1432-2277.2006.00367.x 17018125

[49] Gore JL, Pham PT, Danovitch GM, Wilkinson AH, Rosenthal JT, Lipshutz GS, el at Obesity and outcome following renal transplantation. Am J Transplant. 2006; 6: 357–363. AJT1198 [pii]; 10.1111/j.1600-6143.2005.01198.x 16426321

[50] Cosio FG, Hickson LJ, Griffin MD, Stegall MD, Kudva Y. Patient survival and cardiovascular risk after kidney transplantation: the challenge of diabetes. Am J Transplant. 2008; 8: 593–599. AJT2101 [pii]; 10.1111/j.1600-6143.2007.02101.x 18294155

[51] Revanur VK, Jardine AG, Kingsmore DB, Jaques BC, Hamilton DH, Jindal RM. Influence of diabetes mellitus on patient and graft survival in recipients of kidney transplantation. Clin Transplant. 2001; 15: 89–94. ctr150202 [pii]. 10.1034/j.1399-0012.2001.150202.x 11264633

[52] Cosio FG, Pesavento TE, Kim S, Osei K, Henry M, Ferguson RM. Patient survival after renal transplantation: IV. Impact of post-transplant diabetes. Kidney Int 2002; 62: 1440–1446. kid582 [pii]; 10.1111/j.1523-1755.2002.kid582.x 12234317

[53] Muller E, Kahn D, Mendelson M. Renal Transplantation between HIV-Positive Donors and Recipients. N Engl J Med. 2010; 362: 2336–2337. 10.1056/NEJMc0900837 20554994 PMC5094174

[54] Roland ME, Barin B, Carlson L, Frassetto LA, Terrault NA, Hirose R, et al HIV-Infected Liver and Kidney Transplant Recipients: 1- and 3-Year Outcomes. Am J Transplant. 2008; 8: 355–365. 10.1111/j.1600-6143.2007.02061.x 18093266

[55] Stock PG, Barin B, Murphy B, Hanto D, Diego JM, Light J, et al Outcomes of kidney transplantation in HIV-infected recipients. N Engl J Med. 2010; 363: 2004–2014. 10.1056/NEJMoa1001197 21083386 PMC3028983

[56] Harnett JD, Zeldis JB, Parfrey PS, Kennedy M, Sircar R, Steinmann TI, et al Hepatitis B disease in dialysis and transplant patients. Further epidemiologic and serologic studies. Transplantation. 1987; 44: 369–376. 10.1097/00007890-198709000-00009 2820093

[57] Gane E, Pilmore H. Management of chronic viral hepatitis before and after renal transplantation. Transplantation. 2002; 74: 427–437. 10.1097/00007890-200208270-00001 12352899

[58] Yap DY, Chan TM. Evolution of hepatitis B management in kidney transplantation. World J Gastroenterol. 2014; 20: 468–474. 10.3748/wjg.v20.i2.468 24574715 PMC3923021

[59] Yap DY, Tang CS, Yung S, Choy BY, Yuen MF, Chan TM. Long-term outcome of renal transplant recipients with chronic hepatitis B infection-impact of antiviral treatments. Transplantation. 2010; 90: 325–330. 10.1097/TP.0b013e3181e5b811 20562676

[60] Denhaerynck K, Steiger J, Bock A, Schäfer-Keller P, Köfer S, Thannberger N, el at Prevalence and Risk Factors of Non-Adherence with Immunosuppressive Medication in Kidney Transplant Patients. Am J Transplant. 2007; 7: 108–116. 10.1111/j.1600-6143.2006.01611.x 17109727

[61] Cosio FG, Falkenhain ME, Pesavento TE, Yim S, Alamir A, Henry ML, el at Patient survival after renal transplantation: II. The impact of smoking. Clin Transplant. 1999; 13: 336–341. 10.1034/j.1399-0012.1999.130410.x 10485376

[62] Hurst FP, Altieri M, Patel PP, Jindal TR, Guy SR, Sidawy AN, el at Effect of Smoking on Kidney Transplant Outcomes: Analysis of the United States Renal Data System. Transplantation 2011; 92: 1101–1107. 10.1097/TP.0b013e3182336095 21956202

[63] Moosa MR. Impact of age, gender and race on patient and graft survival following renal transplantation—developing country experience. S Afr Med J. 2003; 93: 689–695. 14635558

[64] Dogan E, Erkoc R, Sayarlioglu H, Durmus A, Topal C (2005) Effects of late referral to a nephrologist in patients with chronic renal failure. Nephrology. 2003; 10: 516–519. 10.1111/j.1440-1797.2005.00433.x 16221105

[65] Smart NA, Titus TT. Outcomes of Early versus Late Nephrology Referral in Chronic Kidney Disease: A Systematic Review. Am J Med. 2011; 124: 1073–1080. 10.1016/j.amjmed.2011.04.026 22017785

[66] Maluka S, Kamuzora P, San SM, Byskov J, Olsen OE, Shayo E, el at Decentralized health care priority-setting in Tanzania: evaluating against the accountability for reasonableness framework. Soc Sci Med. 2010; 71: 751–759. S0277-9536(10)00384-9 [pii]; 10.1016/j.socscimed.2010.04.035 20554365

[67] Byskov J, Bloch P, Blystad A, Hurtig AK, Fylkesnes K, Kamuzora P, el at Accountable priority setting for trust in health systems—the need for research into a new approach for strengthening sustainable health action in developing countries. Health Res Policy Syst. 2009; 7: 23 1478-4505-7-23 [pii]; 10.1186/1478-4505-7-23 19852834 PMC2777144

[68] Norheim OF. Ethical priority setting for universal health coverage: challenges in deciding upon fair distribution of health services. BMC Med. 2016; 14: 75 10.1186/s12916-016-0624-4 [pii]. 27170046 PMC4864904

[69] Norheim OF, Baltussen R, Johri M, Chisholm D, Nord E, Brock D, el at Guidance on priority setting in health care (GPS-Health): the inclusion of equity criteria not captured by cost-effectiveness analysis. Cost Eff Resour Alloc. 2014; 12: 18 10.1186/1478-7547-12-18 25246855 PMC4171087

[70] Moosa MR. Kidney transplantation in developing countries In: Morris PJ, Knechtle SJ, editors. Kidney transplantation: Principles and practice. London: Elsevier Saunders 2014; pp. 643–675.

[71] Vachharajani TJ, Moist LM, Glickman MH, Vazquez MA, Polkinghorne KR, Lok CE, el at Elderly patients with CKD—dilemmas in dialysis therapy and vascular access. Nat Rev Nephrol. 2014; 10: 116–122. 10.1038/nrneph.2013.256 24296629

[72] Carson RC, Juszczak M, Davenport A, Burns A. Is maximum conservative management an equivalent treatment option to dialysis for elderly patients with significant comorbid disease? Clin J Am Soc Nephrol. 2009; 4: 1611–1619. 10.2215/CJN.00510109 19808244 PMC2758251

[73] Daniels N. Accountability for reasonableness. BMJ. 2000; 321: 1300–1301. 10.1136/bmj.321.7272.1300 11090498 PMC1119050

