# Correction: Use of the 'Accountability for Reasonableness' Approach to Improve Fairness in Accessing Dialysis in a Middle-Income Country

**DOI:** 10.1371/journal.pone.0168017

**Published:** 2016-12-09

**Authors:** Mohammed Rafique Moosa, Jonathan David Maree, Maxwell T. Chirehwa, Solomon R. Benatar

The images for Figs 1 and 2 are incorrectly switched. The image that appears as Fig 1 should be Fig 2, and the image that appears as Fig 2 should be Fig 1. The figure captions appear in the correct order.

**Fig 1:**
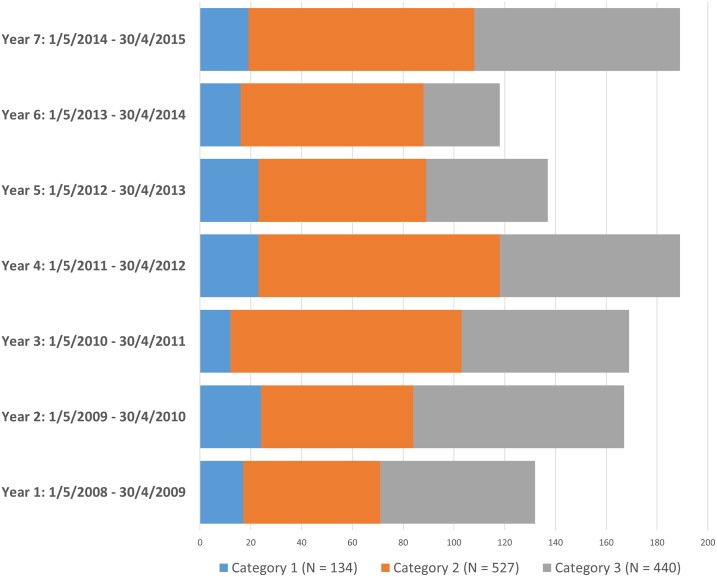
The total number of patients assessed over the period of the study within each assessment category. The majority of patients were assessed as Category 2, which meant that they could be offered treatment only if facilities were available at the time they required dialysis.

**Fig 2:**
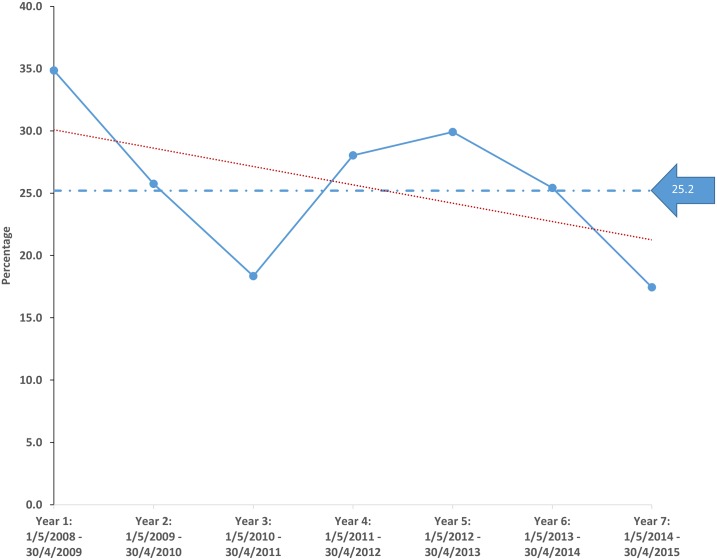
Patient treatment acceptance rates over the seven year time period. The overall acceptance rate was 25.2% (stippled line) with the trend being downward (dotted line). Acceptance almost halved in the seventh year compared to the first, from 34.8% to 17.5%. The fluctuations in numbers with increases in Years 4 and 5 were related to slight expansions in the renal replacement program—the capped number of patients we were allowed to treat was increased from 100 to 120.
